# Ethoxyquin – Determination of 2,2,4-trimethyl-6(2H)-quinolinone in urine by UPLC-MS/MS: Biomonitoring Method – Translation of the German version from 2026

**DOI:** 10.34865/bi9153e11_2or

**Published:** 2026-06-30

**Authors:** Gerhard Scherer, Nikola Pluym, Max Scherer, Nadine Rögner, Markus Stoeckelhuber, Thomas Jäger, Thomas Göen, Andrea Hartwig, MAK Commission

**Affiliations:** 1 ABF Analytisch-biologisches Forschungslabor GmbH Semmelweisstraße 5 82152 Planegg Germany; 2 BASF SE, Corporate Health Management Carl-Bosch-Straße 38 67056 Ludwigshafen Germany; 3 Friedrich-Alexander-Universität Erlangen-Nürnberg, Institute and Outpatient Clinic of Occupational, Social, and Environmental Medicine Henkestraße 9–11 91054 Erlangen Germany; 4 Institute of Applied Biosciences, Department of Food Chemistry and Toxicology, Karlsruhe Institute of Technology (KIT) Adenauerring 20a, Building 50.41 76131 Karlsruhe Germany; 5 Permanent Senate Commission for the Investigation of Health Hazards of Chemical Compounds in the Work Area, Deutsche Forschungsgemeinschaft, Kennedyallee 40, 53175 Bonn, Germany. Further information: Permanent Senate Commission for the Investigation of Health Hazards of Chemical Compounds in the Work Area | DFG

**Keywords:** ethoxyquin, EQI, hydroxyquin, biomonitoring, urine, UPLC-MS/MS

## Abstract

The working group “Analyses in Biological Materials” of the German Senate Commission for the Investigation of Health Hazards of Chemical Compounds in the Work Area (MAK Commission) developed and verified this biomonitoring method for the determination of the most important urinary metabolite of ethoxyquin – 2,2,4‑trimethyl‑6(2H)-quinolinone (EQI). Ethoxyquin is a quinoline-based synthetic antioxidant. Its use as active substance in pesticides was prohibited in 2011. Nevertheless, it continues to be used as a feed additive and is mainly introduced into the environment by feeding treated fishmeal to fish in aquaculture. A human metabolism study showed that ethoxyquin is first metabolised to 1,2‑dihydro-2,2,4‑trimethyl-6‑quinolinol (hydroxyquin) and is then further oxidised to the more stable EQI. In the biomonitoring method presented here, the glucuronide of hydroxyquin in the urine sample is enzymatically hydrolysed using *β*‑glucuronidase from *E. coli*. Thereafter, hydroxyquin oxidises spontaneously to EQI. The hydrolysate is subject to salt-assisted liquid-liquid extraction (SALLE) with ethyl acetate. Analysis is performed by UPLC‑MS/MS after positive electrospray ionisation (ESI+). Calibration is performed using standards prepared in pooled urine and processed analogously to the samples. EQI‑D_10_ is applied as an internal standard. The method provides reliable and accurate analytical results, as shown by the good precision data with standard deviations no greater than 6%. Good accuracy data were obtained with mean relative recoveries in the range of 103–110%. The method is both selective and sensitive, whereby a quantitation limit of 0.03 μg EQI/l was achieved.

## 1 Characteristics of the method

### Matrix and analytical principle

**Table:** 

**Matrix**	Urine
**Analytical principle**	Ultra-high-performance liquid chromatography with tandem mass spectrometry (UPLC‑MS/MS)

### Parameter and corresponding hazardous substance

**Table:** 

Hazardous substance	CAS No.	Parameter	CAS No.
Ethoxyquin	91-53-2	2,2,4‑Trimethyl-6(2H)‑quinolinone (EQI)	4071-18-5

### Reliability criteria

#### 2,2,4‑Trimethyl-6(2H)‑quinolinone (EQI)

**Table:** 

Within-day precision:	Standard deviation (rel.)	*s_w_* = 5.72%, 0.51%, 2.28%, or 1.71%
	Prognostic range	*u* = 15.8%, 1.42%, 6.33%, or 4.75%
	at a spiked concentration of 0.03 μg, 0.1 μg, 1.0 μg, or 10 μg EQI per litre of urine and n = 5 determinations	
Day-to-day precision:	Standard deviation (rel.)	*s_w_* = 5.34%, 3.53%, 3.13%, or 3.44%
	Prognostic range	*u* = 23.0%, 15.2%, 13.5%, or 14.8%
	at a spiked concentration of 0.03 μg, 0.1 μg, 1.0 μg, or 10 μg EQI per litre of urine and n = 3 determinations	
Accuracy:	Recovery (rel.)	*r* = 106%, 107%, 110%, or 103%
	at a spiked concentration of 0.03 μg, 0.1 μg, 1.0 μg, or 10 μg EQI per litre of urine and n = 3 determinations	
Limit of detection:	0.01 μg EQI per litre of urine	
Limit of quantitation:	0.03 μg EQI per litre of urine	

## 2 General information on ethoxyquin

Ethoxyquin (6‑ethoxy-2,2,4‑trimethyl-1,2‑dihydroquinoline) is a synthetic antioxidant of the quinoline class. Ethoxyquin was permitted for use as a biocidal active substance in the European Union. The use of ethoxyquin in pesticides, however, has been banned in the European Union since the end of 2011. (European Commission 2011). Ethoxyquin was further used as a feed additive to extend the shelf life of feed products and to prevent spontaneous combustion in the transport of fishmeal by sea (EFSA FEEDAP Panel 2015). Since there is still no comprehensive exposure assessment for ethoxyquin, its authorisation as a feed additive was suspended by the European Commission as of September 30, 2019 (European Commission 2017). In 2022, the EFSA re-evaluated ethoxyquin as a feed additive (EFSA FEEDAP Panel et al. 2022). Ethoxyquin is synthesised from *p*‑phenitidine (4‑ethoxyaniline), which is categorised as a potential mutagen and is present in the product at concentrations of < 2.5 mg/kg ethoxyquin. Due to a lack of data on the presence of *p*‑phenitidine in food products of animal origin, ethoxyquin could not be conclusively evaluated with respect to consumer safety. However, it was recommended that inhalation exposure to ethoxyquin be kept to a minimum due to this contamination. Moreover, in 2022, an implementing regulation entered into force which denies the authorisation of ethoxyquin as a feed additive in the functional group “antioxidants” within the European Union (European Commission 2022). Ethoxyquin has not yet been evaluated by the MAK Commission.

Ethoxyquin enters the environment primarily through fish farms where edible fish, such as salmon, are fed fishmeal containing ethoxyquin. There are no limit values for any species of fish. In a 2016 study conducted by Greenpeace, ethoxyquin concentrations of up to 881 μg/kg were detected in 54 fish products from German supermarkets (Greenpeace 2016). Ethoxyquin may be converted into a dimer in fishmeal as well as in fishes themselves. This ethoxyquin dimer accumulates primarily in fatty tissue (Ørnsrud et al. 2011).

The metabolism of ethoxyquin has been comprehensively investigated, particularly in aquatic organisms. In fish, the substance is mainly metabolised to 1,2‑dihydro-2,2,4‑trimethyl-6‑quinolinol (hydroxyquin) via O‑deethylation at C6 followed by sulfate or glucuronide conjugation. The oxidation of hydroxyquin to 2,2,4‑trimethyl-6(2H)‑quinolinone (EQI) has been detected as well. In addition, hydroxylation and glucuronidation at C8 have been observed as well as epoxidation between C3 and C4 and dimerisation (Berdikova Bohne et al. 2006, 2007, 2008; Burka et al. 1996; Kranawetvogl and Elsinghorst 2019). For a long time, the metabolism of ethoxyquin in humans was largely unknown. Important metabolites and their toxicokinetics and metabolic conversion factors were investigated in a small metabolism study with five volunteers as part of a cooperative project between the German Federal Ministry for the Enivronment (*Bundesumweltministerium*, BMU) and the German Chemical Industry Association (*Verband der chemischen Industrie*, VCI) for the development of biomonitoring methods (Stoeckelhuber et al. 2020). The metabolism study involved a single oral dose of 0.005 mg ethoxyquin/kg body weight. EQI and the glucuronide of hydroxyquin were identified as the main metabolites of ethoxyquin in the test subjects’ urine samples. Hydroxyquin, which is released via enzymatic hydrolysis, is rapidly oxidised to EQI. Ascorbic acid was added to prevent this oxidation but is largely removed by the later extraction step, such that 6‑OH‑EQ is quantitatively oxidised to EQI after performed extraction. Further oxidation to the *N*‑oxide has not been observed. As part of method development, special care was taken to shift the existing equilibrium entirely in favour of EQI. After complete sample work-up, only trace amounts of 6‑OH‑EQ (below the quantitation limit) were detected in the samples. Ethoxyquin itself was found only in small amounts (< 5% of the ethoxyquin dose) and only in the highly concentrated urine samples of the volunteers. Within 48 h, 28.5% of the orally administered ethoxyquin dose was excreted with the urine in the form of EQI. Ethoxyquin dimers could not be detected in any samples from the metabolism study.

**Fig. 1:**
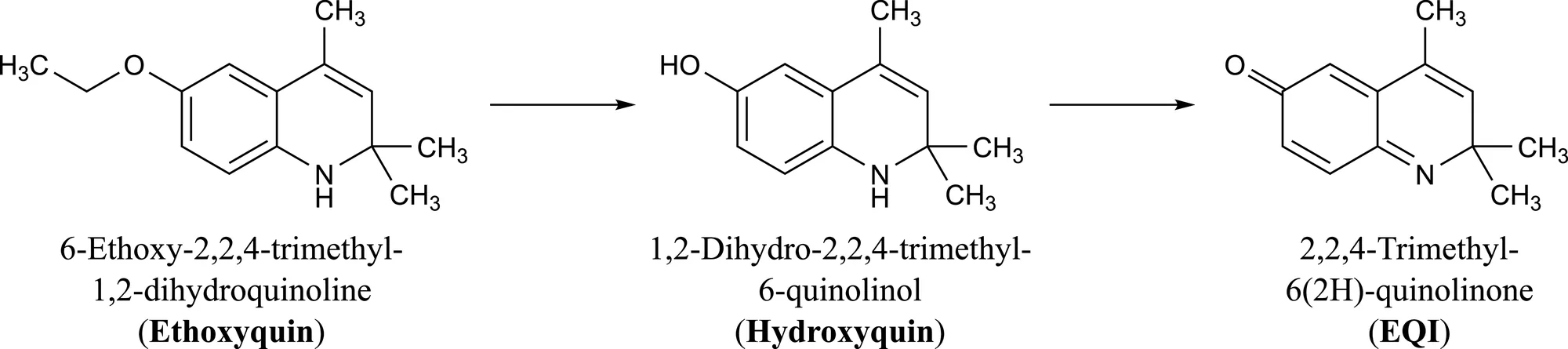
Postulated metabolism of ethoxyquin in humans

Figure 1 depicts the structural formula and the postulated metabolism scheme of ethoxyquin in humans.

To collect data for the metabolism study, Stoeckelhuber et al. (2020) developed a biomonitoring method that enables the measurement of ethoxyquin exposure in the general population. In this method, hydroxyquin is oxidised to the more stable EQI during sample preparation, such that both metabolites are determined together as EQI in urine. Table 1 provides representative background concentrations of EQI in urine from the German general population.

**Tab. 1:** EQI in urine from the general population without occupational exposure

Study collective (country; n; sample)	QF [%]	LOD [μg/l]	LOQ [μg/l]	Mean (SD) [μg/l]	GM [μg/l]	Median [μg/l]	P95 [μg/l]	Min–Max [μg/l]	References
Germany; 53; spot urine	79	0.01	0.03	0.072^a)^ (0.243)^a)^	n. s.	0.015^a)^	n. s.	0.015–1.73	Stoeckelhuber et al. 2020
Germany; 250; 24‑h urine	91	0.01	0.03	0.858^a)^	0.173^a)^	0.16^a)^	1.34^a)^	0.015–104	Pluym et al. 2023

GM: geometric mean; LOD: limit of detection; LOQ: limit of quantitation; n: sample size; n. s.: not stated; P95: 95th percentile; QF: quantitation frequency (measured values above LOQ); SD: standard deviation

^a)^ calculated as LOQ/2 for values below the LOQ

## 3 General principles

The method herein described is used to determine the ethoxyquin metabolite EQI in urine. The ethoxyquin metabolite hydroxyquin is present in urine almost exclusively as a glucuronide. After enzymatic hydrolysis with *β‑*glucuronidase from *E. coli*, the released hydroxyquin is spontaneously oxidised to the more stable EQI. Prior to hydrolysis, ascorbic acid is added to the samples as an antioxidant in order to stabilise EQI. Moreover, to assess the efficacy of the hydrolysis step, 4-methylumbelliferyl-*β*‑D‑glucuronide (MUG) is added to each sample prior to hydrolysis. If enzymatic hydrolysis is successful, MUG is deconjugated to 4-methylumbelliferone (MU) and detected by mass spectrometry after chromatographic separation. The hydrolysate is subjected to salt-assisted liquid-liquid extraction (SALLE) with ethyl acetate. The analysis is performed using UPLC‑MS/MS after positive electrospray ionisation (ESI+). Calibration is performed using calibration standards prepared in pooled urine and processed analogously to the samples. EQI‑D_10_ is used as internal standard.

## 4 Equipment, chemicals, and solutions

### 4.1 Equipment

- UPLC system comprised of a high-pressure pump (LC‑20AB), an ultra-pressure pump (LC‑30AD), a system controller (CBM‑20A), a degasser (DGU‑20A5R), a column oven (CTO‑20AC), and an autosampler (SIL‑30ACMP) (e.g. Shimadzu Deutschland GmbH, Duisburg, Germany)
- Tandem-quadrupole mass spectrometer (e.g. QTRAP^®^ 6500+, AB SCIEX Germany GmbH, Darmstadt, Germany) with data-evaluation software (e.g. Multiquant V.3.0.2, AB SCIEX Germany GmbH, Darmstadt, Germany)
- Nitrogen generator (e.g. NGM 11‑s, cmc Instruments GmbH, Eschborn, Germany)
- HPLC column (e.g. No. 186002352ivd, ACQUITY UPLC BEH C18 1.7 μm, 2.1 × 100 mm, Waters GmbH, Eschborn, Germany)
- Water-purification system (e.g. Sartorius arium^®^, Sartorius AG, Göttingen, Germany)
- Pipettes: 1–10 μl, 10–100 μl, 100–1000 μl, 1000–5000 μl (e.g. Varipettes^®^, Eppendorf AG, Hamburg, Germany)
- Multipette^®^ (e.g. Eppendorf AG, Hamburg, Germany)
- 4‑ml threaded glass bottles (e.g. No. 130400, BGB Analytik Vertrieb GmbH, Rheinfelden, Germany) with screw caps (e.g. No. 2.301158, Klaus Ziemer GmbH, Langerwehe, Germany)
- 100‑ml amber glass bottles (e.g. No. 704012 BRAND GmbH + CO KG, Wertheim, Germany)
- Microvials with inserts (e.g. Klaus Ziemer GmbH, Langerwehe, Germany)
- Various volumetric flasks (e.g. Schott AG, Mainz, Germany)
- pH meter with pH electrode (e.g. Type CG 842, Schott AG, Mainz, Germany)
- Centrifuge (e.g. Rotixa KS, Andreas Hettich GmbH & Co. KG, Tuttlingen, Germany)
- Incubator with shaking apparatus (e.g. Incucell 111 with shaker GFL 3005, MMM Medcenter Einrichtungen GmbH, Planegg, Germany)
- Analytical balance (e.g. Sartorius AG, Göttingen, Germany)
- Multi-tube vortex mixer (e.g. VWR International GmbH, Darmstadt, Germany)
- Vortex shaker (e.g. VWR International GmbH, Darmstadt, Germany)
- Crimper (e.g. Klaus Ziemer GmbH, Langerwehe, Germany)
- Urine cups (e.g. VWR International GmbH, Darmstadt, Germany)

### 4.2 Chemicals

Unless otherwise specified, all chemicals must be a minimum of *pro analysi* grade.

- Acetonitrile, LiChrosolv^®^ (e.g. No. 10003, Supelco, Merck KGaA, Darmstadt, Germany)
- Ascorbic acid (e.g. No. A5960, Merck KGaA, Darmstadt, Germany)
- Ethyl acetate (e.g. No. 2219, Th. Geyer GmbH & Co. KG, Renningen, Germany)
- Formic acid, 0.1% in acetonitrile (e.g. No. 2645, Th. Geyer GmbH & Co. KG, Renningen, Germany)
- Formic acid, 99% (e.g. No. 069141, Biosolve B.V., Valkenswaard, Netherlands)
- Glacial acetic acid, > 99% (e.g. No. 33252, Alfa Aesar, Thermo Fisher Scientific^TM^, Life Technologies GmbH, Darmstadt, Germany)
- *β*‑Glucuronidase from *E. coli* (500 kU; 250 kU/ml) (e.g. No. E‑BGLAEC, Megazyme Ltd., Bray, Ireland)
- Magnesium sulfate (e.g. No. M7506, Merck KGaA, Darmstadt, Germany)
- Methanol (e.g. No. 1428, Th. Geyer GmbH & Co. KG, Renningen, Germany)
- 4‑Methylumbelliferyl-*β*‑D‑glucuronide dihydrate (e.g. No. sc-284360, Santa Cruz Biotechnology Inc., Dallas, USA)
- Sodium chloride (e.g. No. S7653, Merck KGaA, Darmstadt, Germany)
- Sodium hydroxide pellets (e.g. No. 106469, Merck KGaA, Darmstadt, Germany)
- 2,2,4‑Trimethyl-6(2H)‑quinolinone (EQI) (e.g. in-house synthesis, ABF GmbH, Munich, Germany, see Supplementary information to Stoeckelhuber et al. 2020)
- 2,2,4‑Trimethyl-6(2H)‑quinolinone‑D_10_ (EQI‑D_10_) (e.g. custom synthesis, Synthèse AptoChem Inc., Montreal, Canada)
- Ultra-pure water (e.g. Sartorius arium^®^, Sartorius AG, Göttingen, Germany)

### 4.3 Solutions

- Ascorbic acid solution (100 g/l)
  Exactly 10 g of ascorbic acid are weighed into a 100‑ml volumetric flask and dissolved in a little ultra-pure water. The volumetric flask is then made up to the mark with ultra-pure water.

The solution is stable at room temperature for three months.

- Acetate buffer (1 mol/l, pH = 5.1)
  About 500 ml of ultra-pure water are placed in a 1000‑ml volumetric flask and 57 ml of acetic acid (> 99%) are added. The pH value is adjusted to pH = 5.1 using sodium hydroxide pellets. The volumetric flask is then made up to the mark with ultra-pure water.

The solution is stable at room temperature for three months.

- *β*‑glucuronidase suspension (250 kU/ml)
  A bottle (2 ml) of *β*‑glucuronidase (500 kU/ml) is diluted with 2 ml of ultra-pure water.

The solution is stable at 4 °C for three months.

- Sodium hydroxide solution (14.3 mol/l or 40%)
  Ultra-pure water is placed in a beaker, in which 57.2 g of sodium hydroxide pellets are then dissolved. The sodium hydroxide solution is then transferred into a 100‑ml volumetric flask and made up to the mark with ultra-pure water.

The solution is stable at room temperature for ten years.

- Eluent A (0.1% formic acid in ultra-pure water)
  About 500 ml of ultra-pure water are placed in a 1000‑ml volumetric flask and 1 ml of formic acid is added. The volumetric flask is then made up to the mark with ultra-pure water.

The solution is stable at room temperature for ten years.

- Eluent B (0.1% formic acid in acetonitrile)
  About 500 ml of acetonitrile are placed in a 1000‑ml volumetric flask and 1 ml of formic acid is added. The volumetric flask is then made up to the mark with acetonitrile.

The solution is stable at room temperature for ten years.

- 4‑Methylumbelliferyl-*β*‑D‑glucuronide (MUG) stock solution (5000 mg/l)
  9.5 mg of MUG dihydrate are weighed into a 5-ml volumetric flask and dissolved in 1724 μl of methanol.

The MUG stock solution is stable at 4 °C for several years.

- MUG working solution (50 mg/l)
  100 μl of the MUG stock solution are pipetted into a 10-ml volumetric flask. The volumetric flask is then made up to the mark with ultra-pure water.

The MUG working solution is stable at 4 °C for several years.

### 4.4 Internal Standard (ISTD)

- ISTD stock solution (1 g/l)
  In a 10-ml volumetric flask, 10.0 mg of EQI‑D_10_ are weighed and dissolved in acetonitrile. The volumetric flask is then made up to the mark with acetonitrile.
- ISTD working solution I (100 mg/l)
  2700 μl of acetonitrile are pipetted to 300 μl of the ISTD stock solution.
- ISTD working solution II (10 mg/l)
  2700 μl of acetonitrile are pipetted to 300 μl of ISTD working solution I.
- ISTD spiking solution (1 mg/l)
  2700 μl acetonitrile are pipetted to 300 μl of ISTD working solution II.

The ISTD stock, working, and spiking solutions are stored in amber glass bottles at −20 °C and are stable under these conditions for at least 18 months.

### 4.5 Calibration standards

- EQI stock solution I (1000 mg/l)
  In a 10-ml volumetric flask, 10.0 mg of EQI are weighed and dissolved in acetonitrile. The volumetric flask is then made up to the mark with acetonitrile.
- EQI stock solution II (100 mg/l)
  2700 μl of acetonitrile are pipetted to 300 μl of EQI stock solution I.
- EQI stock solution III (10 mg/l)
  2700 μl of acetonitrile are pipetted to 300 μl of EQI stock solution II.
- EQI working solution I (1 mg/l)
  2700 μl of acetonitrile are pipetted to 300 μl of EQI stock solution III.
- EQI working solution II (100 μg/l)
  2700 μl of acetonitrile are pipetted to 300 μl of EQI working solution I.
- EQI working solution III (10 μg/l)
  2700 μl of acetonitrile are pipetted to 300 μl of EQI working solution II.
- EQI working solution IV (1 μg/l)
  2700 μl of acetonitrile are pipetted to 300 μl of EQI working solution III.

The stock and working solutions are stored in amber glass bottles at −20 °C. EQI stock solution I is stable for at least 18 months at −20 °C.

Pooled urine with the lowest possible analyte concentrations is used for calibration. To prepare the calibration standards, the urine is mixed with the EQI working solutions and the ISTD spiking solution according to the pipetting scheme shown in Table 2. Unspiked pooled urine is included to estimate the blank value.

**Tab. 2:** Pipetting scheme for the preparation of calibration standards for the determination of EQI in urine

Calibration standard	EQI working solution IV [μl]	EQI working solution III [μl]	EQI working solution II [μl]	EQI working solution I [μl]	ISTD spiking solution [μl]	Pooled urine [μl]	Analyte concentration [μg/l]
S00	–	–	–	–	–	3000	0
S0	–	–	–	–	10	2990	0
S1	90	–	–	–	10	2900	0.03
S2	–	15	–	–	10	2975	0.05
S3	–	30	–	–	10	2960	0.1
S4	–	60	–	–	10	2930	0.2
S5	–	–	15	–	10	2975	0.5
S6	–	–	30	–	10	2960	1
S7	–	–	60	–	10	2930	2
S8	–	–	–	15	10	2975	5
S9	–	–	–	30	10	2960	10
S10	–	–	–	60	10	2930	20

## 5 Specimen collection and sample preparation

### 5.1 Specimen collection

Urine samples are collected in sealable urine cups and stored at −20 °C until sample preparation.

### 5.2 Sample preparation

The frozen urine samples are thawed at room temperature and thoroughly mixed before analysis. Aliquots of 3 ml urine are transferred into 4‑ml threaded glass bottles, then 10 μl of ascorbic acid solution, 10 μl of ISTD spiking solution, 0.5 ml of acetate buffer, 10 μl of MUG working solution, and 10 μl of *β‑*glucuronidase suspension are added. The samples are mixed well and are then shaken for one hour in an incubator at 37 °C on a vortex shaker at 100 rpm.

The hydrolysed samples are carefully transferred into new 4‑ml vials which contain 0.8 g of magnesium sulfate and 0.2 g of sodium chloride. Subsequently, 100 μl of sodium hydroxide solution (14.3 mol/l) and 400 μl of ethyl acetate are added by pipetting. Using a multi-tube vortex mixer, the samples are mixed for 10 min at 2500 rpm and are then centrifuged for 10 min at 2500 × *g*. Samples with insufficient phase separation are shaken and centrifuged a second time. 100 μl of the organic phase are transferred into a microvial with insert, and applied for analysis by UPLC‑MS/MS.

## 6 Operational parameters

Analytical determination was carried out using a device configuration comprised of a UPLC system coupled to a tandem mass spectrometer.

### 6.1 Liquid chromatography

**Table:** 

Separation column:	ACQUITY UPLC BEH C18 (2.1 mm × 100 mm, 1.7 μm)
Column temperature:	isothermal, 40 °C
Autosampler temperature:	10 °C
Injection volume:	5 μl
Eluent:	A: water + 0.1% formic acid
	B: acetonitrile + 0.1% formic acid
Flow rate:	0.5 ml/min
Pressure:	600–700 bar
Gradient programme:	see Table 3

**Tab. 3:** Gradient programme for the determination of EQI in urine

Time [min]	Eluent A [%]	Eluent B [%]
0.00	90	10
1.00	90	10
4.00	10	90
6.00	10	90
6.01	90	10
8.00	90	10

### 6.2 Tandem mass spectrometry

**Table:** 

Ionisation:	Positive electrospray ionisation (ESI+)
Further settings:	see Table 4

**Tab. 4:** Mass transitions, retention times and other parameter-specific settings for the determination of EQI in urine

Analyte or ISTD	Retention time [min]	Mass transition (*m/z*)	Declustering potential [V]	Collision energy [V]	Collision-cell exit potential [V]
MU	2.9	177.1 → 76.9^a)^	36	49	10
EQI	3.4	188.0 → 173.1^a)^	13	21	10
	3.4	188.0 → 145.1	13	35	10
EQI‑D_10_	3.3	198.0 → 152.1^a)^	13	35	10

EQI: 2,2,4‑trimethyl-6(2H)‑quinolinone; ISTD: internal standard; MU: 4-methylumbelliferone

^a)^ quantifier

The device-specific parameters must be determined and set individually by the user for the system used. The device-specific parameters indicated in this section have been determined and optimised for the system used during method development.

**Fig. 2:**
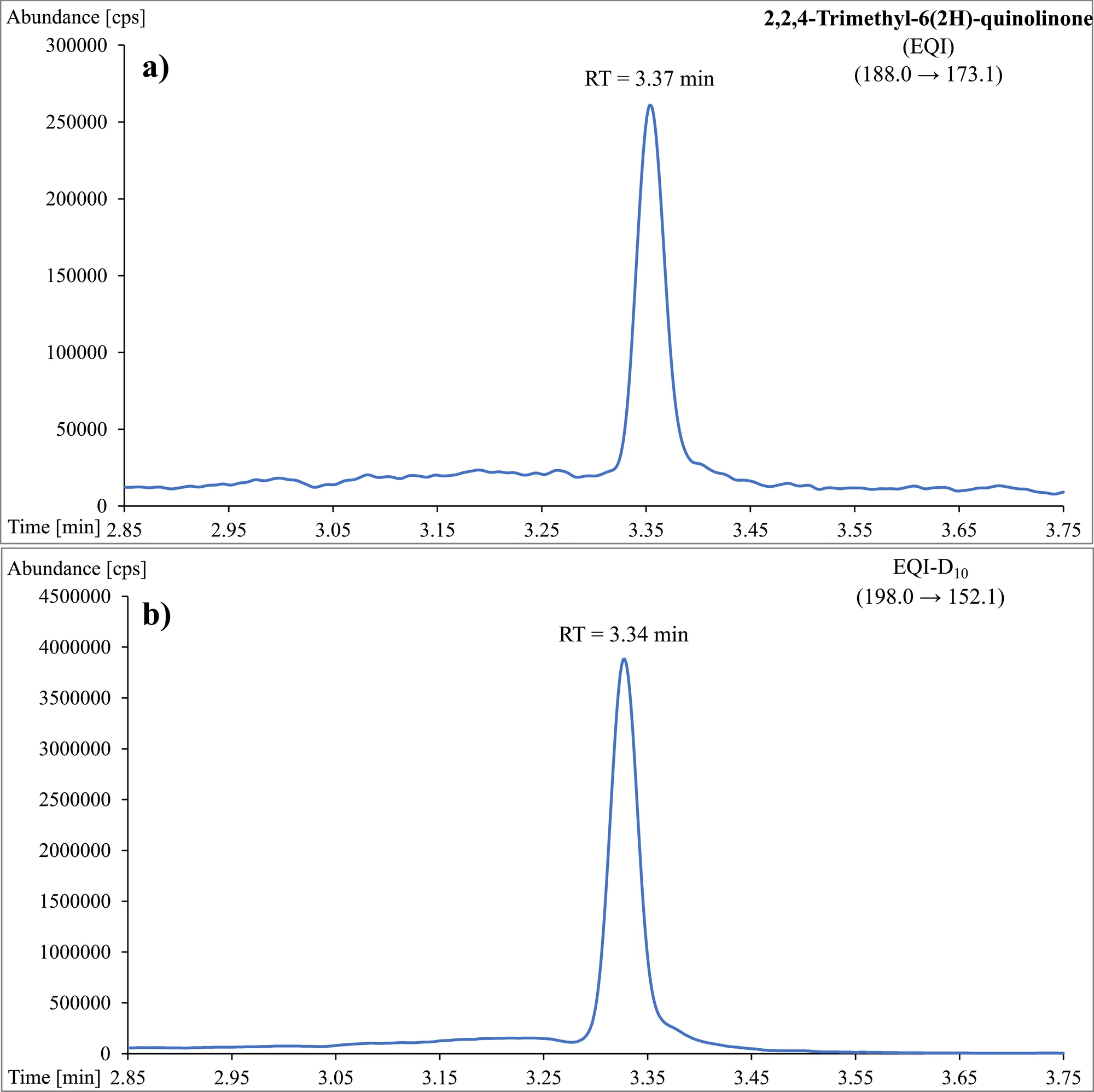
Chromatogram of a urine sample spiked with 0.1 μg EQI per litre

Figure 2 shows a representative chromatogram of a urine sample spiked with 0.1 μg EQI/l.

## 7 Analytical determination

For the analytical determination of the urine samples prepared in accordance with Section 5, an aliquot of 5 μl is injected into the UPLC‑MS/MS system via the autosampler. After chromatographic separation, the analyte and ISTD are identified based on their retention times and characteristic mass transitions (see Table 4). Pooled urine as a blank value (calibration standards S00 and S0) and quality-control samples are included in each analytical run.

The mass transfer of MU (see Table 4) is used to verify the efficiency of the enzymatic hydrolysis. If hydrolysis is successful, the signal should be at least 2 × 10^6^ (abundance). If a significantly smaller signal or even no signal is detectable, it can be assumed that that sample was not sufficiently hydrolysed, requiring renewed work-up and analysis.

## 8 Calibration

The calibration standards prepared as described in Section 4.5 are processed analogously to the urine samples – without further addition of ISTD – (see Section 5) and analysed according to Sections 6 and 7. The calibration curve is generated by linear regression using 1/y weighting of the peak-area ratios of EQI/EQI‑D_10_ against the concentrations of the respective calibration standards. Under the analytical conditions described, the calibration curve is linear in the concentration range of 0.03 to 20 μg/l urine. Samples with higher analyte concentrations must be newly analysed after dilution with ultra-pure water. The corresponding dilution factor is taken into account when calculating analyte concentrations. To verify that samples were measured correctly after dilution, three different urine samples were spiked with 30 μg EQI per litre. These samples were processed and analysed after dilutions of 1 ∶ 3, 1 ∶ 6 and 1 ∶ 30. Five determinations were carried out for each urine sample and dilution level, where the determined accuracies were found to be within the acceptance range of 85–115%.

**Fig. 3:**
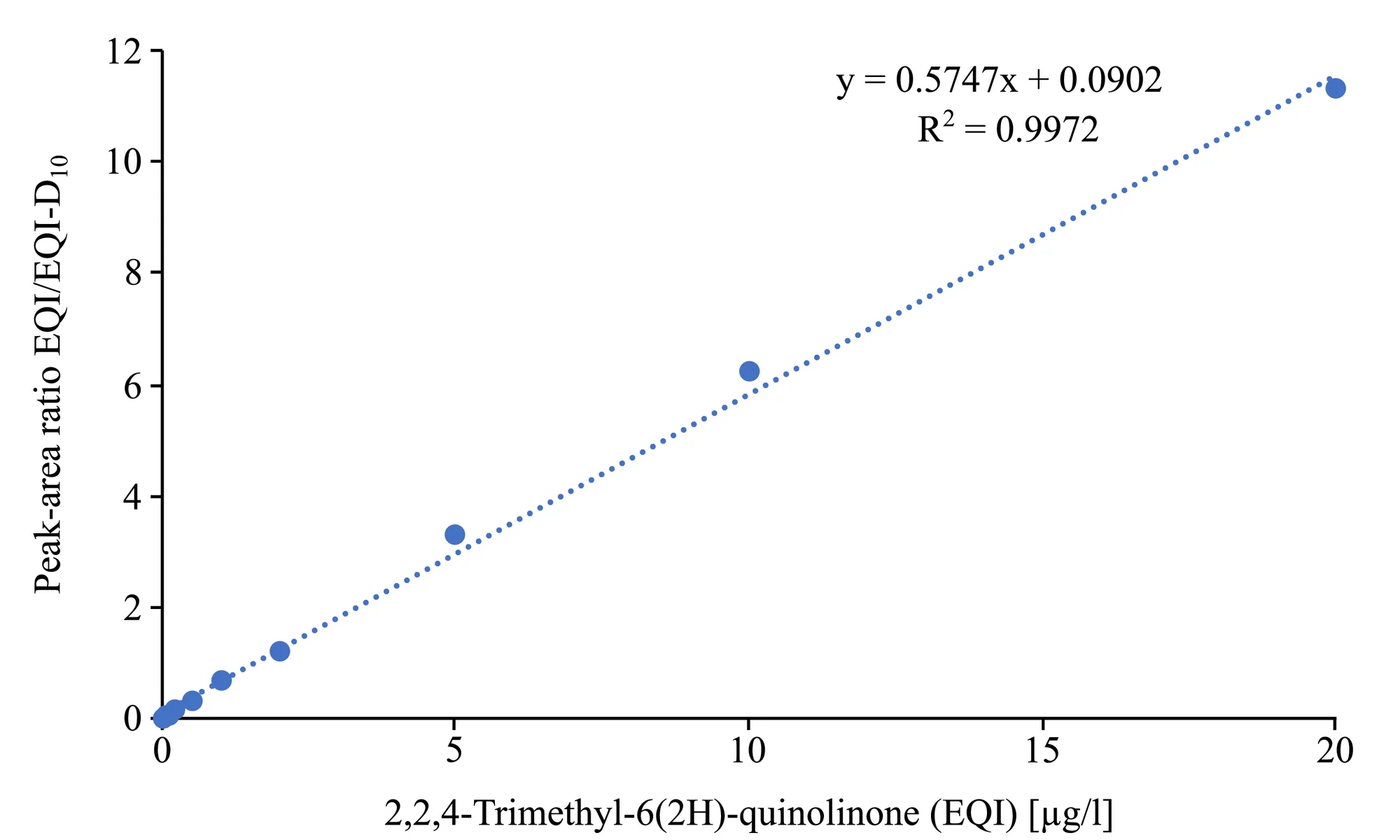
Calibration curve for the determination of EQI in urine

A representative calibration curve for the determination of EQI in urine is shown in Figure 3.

## 9 Calculation of analytical results

The analyte concentration of the respective urine sample is calculated using the calibration function of the corresponding analytical run (see Section 8). The peak‑area ratio of analyte to ISTD is entered into the calibration function to obtain the respective analyte concentration in μg/l urine.

## 10 Standardisation and quality control

Quality assurance of the analytical results is carried out as stipulated in the guidelines of the *Bundesärztekammer* (German Medical Association) and in a general chapter published by the Commission (Bader et al. 2010; Bundesärztekammer 2023).

To check precision, at least one urine with a known analyte concentration is included as quality-control sample in each analytical run. As such quality-control material is not commercially available, it must be prepared in the in-house laboratory. For this purpose, human urine is spiked with a defined amount of EQI, aliquoted, and stored at −20 °C. Four different control materials were prepared for the validation of this method, whereby Q_1_, Q_2_, Q_3_, and Q_4_ contained concentrations of 0.03 μg EQI/l, 0.10 μg EQI/l, 1.00 μg EQI/l, and 10.0 μg EQI/l, respectively.

## 11 Evaluation of the method

The reliability of the method was confirmed by comprehensive validation as well as by replication and verification of the method in a second, independent laboratory. The approach to method validation (consistent with the criteria of the AibM working group of the DFG (Bader et al. 2010) as well as FDA guidelines (FDA 2018)) and the validation data are described in the following sections.

### 11.1 Precision

Within-day precision was determined by processing and analysing the quality-control materials (see Section 10) five times in parallel. The within-day precision data are given in Table 5.

**Tab. 5:** Within-day precision for the determination of EQI in urine (n = 5)

Analyte	Spiked concentration [μg/l]	Measured concentration [μg/l]	Standard deviation (rel.) *s_w_* [%]	Prognostic range *u* [%]
EQI	0.03	0.03	5.72	15.8
	0.10	0.11	0.51	1.42
	1.00	1.07	2.28	6.33
	10.0	9.95	1.71	4.75

To determine day-to-day precision, the relative standard deviations were calculated from the mean values of five parallel determinations conducted over three days. The precision data thus obtained are provided in Table 6.

**Tab. 6:** Day-to-day precision for the determination of EQI in urine (n = 3)

Analyte	Spiked concentration [μg/l]	Measured concentration [μg/l]	Standard deviation (rel.) *s_w_* [%]	Prognostic range *u* [%]
EQI	0.03	0.03	5.34	23.0
	0.10	0.11	3.53	15.2
	1.00	1.10	3.13	13.5
	10.0	10.29	3.44	14.8

### 11.2 Accuracy

The relative recoveries of the analyte were calculated from the day-to-day precision data. The data thus obtained are presented in Table 7.

**Tab. 7:** Relative recovery for the determination of EQI in urine (n = 3)

Analyte	Spiked concentration [μg/l]	Mean recovery (rel.) *r* [%]	Range [%]
EQI	0.03	106	102–111
	0.10	107	106–108
	1.00	110	107–112
	10.0	103	99.5–107

### 11.3 Testing preparation-related losses

To test work-up‑related losses, native urine samples with different EQI background levels were spiked with EQI at concentrations of 0.10 μg/l, 1.00 μg/l, and 10.0 μg/l both before and after sample work-up. The samples were processed and measured three times in parallel, yielding mean absolute recoveries of 13.3%, 14.6% and 17.1% (Table 8). These rather low recoveries are due to the fact that only 0.4 ml of ethyl acetate were used to extract the analyte from approximately 3.5 ml of aqueous phase.

**Tab. 8:** Mean absolute recovery for the determination of EQI in urine (n = 3)

Analyt	Spiked concentration [μg/l]	Mean absolute recovery [%]	Range [%]
EQI	0.10	13.3	11.3–14.7
	1.00	14.6	13.2–15.7
	10.0	17.1	16.8–17.3

### 11.4 Stability

To test analyte stability, the short-term stability, freeze-thaw stability, and stability of the analyte after sample preparation were investigated. For the determination of the above parameters, two urine samples were spiked with a low and a high EQI concentration. The results of the stability test are shown in Table 9. The short-term stability of the analyte was determined after a storage duration of about 20 h at room temperature, which is intended to reflect specimen collection and sample preparation. Freeze-thaw stability was determined using one urine aliquot, which was processed and analysed in triplicate directly as well as after six freeze-thaw cycles. The post-preparative stability test provides insight into the stability of the final extracts in the autosampler under real-world measurement conditions. To this end, processed samples were stored in the cooled autosampler at 10 °C for four days, then measured again. The determination of EQI fulfilled all acceptance criteria for stability (accuracy between 85% and 115% of the target value).

**Tab. 9:** Results of the stability tests (n = 3)

Stability tests	Spiked concentration [μg/l]	Measured concentration [μg/l]	Mean relative recovery [%]	Range [%]
Short-term stability: 20 h at RT	0.05	0.05	98.2	86.0–115
	12.17	10.81	88.8	88.4–89.1
Freeze-thaw stability: after 6 cycles	0.05	0.05	99.7	88.0–108
	12.17	12.46	102	97.5–106
Post-preparative stability: 4 d at 10 °C	0.05	0.04	86.3	85.1–87.2
	12.17	12.02	98.7	94.3–103

### 11.5 Limits of detection and quantitation

The limit of quantitation (LOQ) was ascertained by spiking pooled urine, which was then processed and measured five times in parallel on three different days. The accuracy and precision of the determinations served as criteria. For EQI, the individual accuracy values at the LOQ were between 80% and 120%, and for precision, a coefficient of variation of 20% was not exceeded. The LOQ was thus specified to be 0.03 μg EQI/l of urine. The detection limit is calculated by dividing the LOQ by three, yielding 0.01 μg EQI/l of urine (Table 10)*.*

**Tab. 10:** Limit of detection and quantitation for the determination of EQI in urine

Analyte	Limit of detection [μg/l]	Limit of quantitation [μg/l]
EQI	0.01	0.03

### 11.6 Sources of error

During method development, it was found that in the unprocessed urine samples, ethoxyquin itself is broken down to more than 50% within a few days. Moreover, ethoxyquin was only detectable at low concentrations in the urine samples from the metabolism study. As a result, ethoxyquin was determined not to be a suitable biomarker by the method developers, and method validation was only performed for the ethoxyquin metabolite EQI.

Stability testing revealed that EQI in the urine samples is stable for six freeze-thaw cycles (see Section 11.4). Frequent thawing and refreezing should be avoided. For sample work-up, it is necessary to add ascorbic acid to the samples prior to hydrolysis (see Section 5.2) to increase analyte stability.

Due to the volatility of EQI, it was not possible to use a solvent for the SALLE, that would have required to be blown off. Ethyl acetate was therefore chosen as a solvent suitable for the subsequent chromatographic separation (Stoeckelhuber et al. 2020). The small volume of extraction solvent of only 0.4 ml, however, led to substantial analyte losses during sample preparation (see Section 11.3), a problem which was likewise observed during external method verification. Nevertheless, these losses could be readily compensated by the use of a structurally identical, deuterated ISTD, without significantly compromising the sensitivity of the method.

The signal suppression observed during external method verification was also effectively compensated for by the ISTD. Since the method developers did not observe any such matrix effects, it is conceivable that, in addition to the modified chromatographic conditions during verification, the *β*-glucuronidase/arylsulfatase from *Helix pomatia* used by the method verifiers may have played a role as well (see also Stoeckelhuber et al. 2020).

After injecting samples with high analyte concentrations, a carryover in the LOQ range was observed by the developers of the method. Blank samples should therefore be injected after measuring high calibration standards and quality-control samples. If EQI levels near the LOQ are quantified in samples following samples with high analyte concentrations within an analytical run, these samples must be reinjected.

If the assessment of enzymatic cleavage reveals insufficient hydrolysis efficiency, it is recommended to reprocess and newly analyse the sample in question (see Section 7).

## 12 Discussion of the method

The aim of method development was to develop a rapid and selective analytical procedure to determine possible ethoxyquin exposure in the general population (Stoeckelhuber et al. 2020). This method not only presents the UPLC‑MS/MS method for the determination of EQI in urine, but also discusses in detail the collected validation data and provides the findings from comprehensive method validation in a second, independent laboratory. The method has proven to be sensitive, precise, and highly accurate. The LOQ is sufficient to quantify the ethoxyquin metabolite EQI in most urine samples from the general population and thus estimating non-occupational exposure to ethoxyquin.

Instruments used UPLC‑MS/MS system comprised of the modules LC‑20AB (high-pressure pump), LC‑30AD (ultra-pressure pump), CBM‑20A (system controller), DGU‑20A5R (degasser), CTO‑20AC (column oven), and SIL‑30ACMP (autosampler) (Shimadzu Deutschland GmbH, Duisburg, Germany) as well as a QTRAP^®^ 6500+ tandem-quadrupole mass spectrometer (AB SCIEX Germany GmbH, Darmstadt, Germany)
